# Sarcopenia severity and physical fitness levels in nursing home residents and community-dwelling older adults

**DOI:** 10.1007/s11845-026-04467-z

**Published:** 2026-05-29

**Authors:** Büşra Seçkinoğulları Korkusuz, Süleyman Korkusuz, Ferdi Yavuz, Zeliha Özlem Yürük

**Affiliations:** 1 Kızılcahamam Vocational School of Health Services, Department of Therapy and Rehabilitation, Ankara University, Ankara, Turkey; 2 Faculty of Health Sciences, Department of Physical Therapy and Rehabilitation, Atılım University, Ankara, Turkey; 3 Department of Physical Therapy and Rehabilitation, European University of Lefke, TR-10 Mersin, Lefke, Northern Cyprus Turkey; 4 Faculty of Health Sciences, Department of Physiotherapy and Rehabilitation, Başkent University, Ankara, Turkey

**Keywords:** Sarcopenia, Physical Fitness, Community-Dwelling Older Adults, Nursing Home Resident, Ultrasonography

## Abstract

**Background:**

It is important to evaluate the physical fitness and sarcopenia levels of older adults, taking into account their living environment.

**Objective:**

The aim of our study was to compare the sarcopenia severity and physical fitness levels of nursing home residents and community-dwelling older adults using the ISarcoPRM diagnostic algorithm incorporating ultrasonographic measures.

**Methods:**

Nursing home residents (NHR) (*n* = 51) and community-dwelling older adults (CDO) (*n* = 52) were included in this cross-sectional study. Participants’ physical fitness levels were assessed using the Senior Fitness Test (SFT). Sarcopenia severity was assessed using the 5r-Chair Stand Test (5r-CST) and hand grip strength (HGS), as well as the anterior thigh thickness (ATT) and Sonographic Thigh Adjustment Rate (STAR) obtained from ultrasonographic measurements. Participants were defined as nonsarcopenic, sarcopenic, and dynapenic according to the ISarcoPRM diagnostic algorithm of Kara et al.

**Result:**

In our study, it was seen that individuals in NHR and CDO showed a homogeneous distribution in terms of sarcopenia severity (*p* > 0.05). When the groups were compared, the ATT, STAR, HGS and 5r-CST values of NHR were lower than CDO (*p* < 0.05). When comparing the groups, the physical fitness of NHR was lower than that of CDO according to SFT parameters (*p* < 0.05).

**Conclusion:**

The results of our study showed that geriatric individuals living in NHR had higher levels of sarcopenia and lower levels of physical fitness than CDO. We believe that this result is due to the fact that NHR are dependent on caregivers in the institution where they live for their self-care and daily living activities.

**Clinical trial code:**

NCT06394648

## Introduction

Aging is a process that causes physical, physiological, and social decline [1]. Environmental factors also play an essential role in healthy aging. Home is an environment where the individual feels safe and used to live. Community-dwelling older adults have to do daily living and home activities. Some older adults choose to live in nursing homes due to health problems, fear of falling, socialization, or convenience. A nursing home is a new environment that provides control over the health of individuals, reduces domestic responsibilities, and offers more social opportunities [1, 2].

Sarcopenia is a critical health problem among the elderly population characterized by age-related loss of muscle mass and muscle function. Sarcopenia is the reason for physical weakness, limited movements, and mortality [3–6].

There are a few techniques to determine sarcopenia. Measurement of total (non-regional) muscle mass is one of the techniques. Considering only the decrease in total muscle mass to determine sarcopenia may lead to incorrect results. Total muscle mass decreases with aging. However, it is not homogeneous. For example, the anterior thigh and abdominal muscles atrophy earlier than other muscles [7]. Therefore, in the early stages of sarcopenia, loss of lower extremity muscle mass may occur before upper extremity loss and may be responsible for the decrease in physical performance and function. Therefore, simply considering the reduction in total muscle mass may not be sufficient to interpret muscle loss quickly. Because there may be a normal or even compensatory increase in upper extremity muscles [7–10]. Therefore, the use of the ISarcoPRM algorithm, which calculates the STAR by dividing the anterior thigh muscle thickness (mm) by BMI, including physical performance and muscle strength tests, may be reliable in the definition of sarcopenia [5, 11].

Dynapenia, defined as the loss of muscle strength due to aging, is associated with mortality as well as various adverse medical conditions such as dementia and metabolic syndrome. In the aging population, the distribution of fatty tissues changes with increased infiltration of fat into skeletal muscles. It is known that intramuscular lipid accumulation causes loss of muscle strength. Although dynapenia is common in individuals with decreased skeletal muscle mass, i.e., sarcopenic individuals, a certain population develops loss of muscle strength despite having normal skeletal muscle volume [12].

The environment of older adults may affect physical fitness [1]. Therefore, it is important to evaluate the physical fitness and sarcopenia levels of older adults, taking into account their living environment. Kang et al. [13] stated that older adults living in nursing homes do less housework than older adults living in the community. Responsibilities may help maintain physical fitness in community-dwelling older adults. Many older adults choose to live in a nursing home for reasons such as chronic illness, polypharmacy, or increased risk of falls. These factors also lead to deterioration of physical fitness levels in nursing home residents. On the other hand, social and recreational activities in nursing homes can maintain physical fitness. Although there is no clear consensus on this issue in the literature, previous studies have generally compared the functional characteristics of nursing home residents and community-dwelling older adults, such as physical fitness, functionality, balance, and falls. When the literature is examined, there are studies examining the sarcopenia severity of nursing home residents and community-dwelling older adults [2, 14]. The sarcopenia was evaluated with total muscle mass measurement in these studies.

For this reason, the aim of our study was to compare the sarcopenia severity and physical fitness level of nursing home residents and community-dwelling older adults with an updated and new method, ISarcoPRM. The first hypothesis was that sarcopenia severity was different between nursing home residents and community-dwelling older adults. The second hypothesis was that physical fitness level was different between nursing home residents and community-dwelling older adults.

## Methods

### Study design and participants

The study had a cross-sectional design and was registered on ClinicalTrials.gov (NCT06394648). It was carried out in accordance with the Helsinki Declaration and received ethical approval from the Atılım University Ethics Committee for Human Research. A total of 103 older adults participated in the study. The 51 older adults were living in a private nursing home (Nefes Nursing Home) in Ankara. The 52 older adults were living in their home in Cankaya, Ankara. All participants signed an ethics committee-approved informed consent form.

To avoid bias in the analysis results, inclusion and exclusion criteria were carefully determined to ensure that the patients had similar characteristics.

The inclusion criteria for nursing home residents are (1) being between the ages of 65 and 85, (2) getting a score between 24 and 30 on the Mini-Mental State Examination (MMSE), (3) living in a nursing home for at least 6 months, and (4) being able to stand unaided for at least 90 s.

The inclusion criteria for community-dwelling older adults are (1) between the ages of 65 and 85, (2) getting a score between 24 and 30 on the MMSE, and (3) ability to stand unaided for at least 90 s. Exclusion criteria were as follows: (1) inability to communicate verbally (2) known presence of dementia, (3) use of a walking aid, (4) older adults with severe visual impairment, neurological disorders, and/or congestive heart failure (5) for whom exercise is not recommended older adults.

### Sample size

Sample size (G*Power Ver. 3.1) was determined according to the ‘arm curl’ parameter in the study of Glenn et al. [15]. It was determined that there should be at least 43 individuals in the groups with 80% power. Considering the possible data loss, it was planned to include 52 individuals in both groups. After extreme value analysis, the study was completed with 51 individuals living in a nursing home and 52 individuals living in the community.

### Procedures

Nursing home residents (*n* = 51) and community-dwelling older adults (*n* = 52) participated in the study. Physical fitness levels of the participants were evaluated with the Senior Fitness Test. Sarcopenia was assessed with ultrasonographic imaging. Participants were divided into 3 stages according to the Kara et al.‘s ISarcoPRM diagnostic algorithm: non-sarcopenic, sarcopenic, and dynapenic.

#### Physical fitness evaluation

In our study, we utilized the Senior Fitness Test, developed by Rikli and Jones [16], to assess the physical fitness levels of older adults. This test evaluates functional fitness, which refers to the ability to perform activities of daily living (ADLs) safely, independently, and without excessive fatigue. The Senior Fitness Test consists of seven subtests that target various aspects of functional fitness, along with an assessment of body composition.

- **Chair Stand Test (CST-30s)**: The test measured lower extremity strength and endurance. Participants sat in a chair without armrests, feet flat on the floor, and arms crossed. They stood up completely and sat down as many times as possible within 30 s. The number of repetitions was recorded.
- **Arm Curl Test (ACT)**: The test measured upper body strength. Participants sat on a chair without armrests. They hold a weight (2.27 kg for women, 3.63 kg for men) with their dominant arm fully extended. They then curled the weight towards their shoulder for 30 s. The total number of complete curls was recorded.
- **8-Foot Up-and-Go Test (8-FUGT)**: The test assessed dynamic balance and agility. Participants stood up from a chair without armrests and walked as quickly and safely as possible along a straight 8-foot (2.44 m) path. They then turned around a cone placed at the end, walked back to the chair, and sat down. The time taken to complete the entire course was recorded in seconds.
- **2-Minute Step Test (2-MST)**: The test is an alternative to the 6-minute walk test and measures aerobic endurance. The distance from the hip bone (iliac crest) to the middle of the kneecap (patella) was measured to determine a target stepping height. Participants stood next to a wall marked with this height. They then stood up and down as quickly and safely as possible for two minutes, ensuring each step reached the marked height. The total number of valid steps was recorded.
- **Chair Sit-and-Reach Test (CSRT)**: The modified sit-and-reach test assessed hamstring flexibility. Participants sat on the edge of a chair and extended one leg straight out with the heel flat and ankle at a 90-degree angle. The other leg was bent at a 90-degree angle. Participants reached forward with the same-side hand towards the toes of the extended leg. The distance between the fingertips and the toes was recorded: a negative score if they could not reach, zero if they touched, and a positive score if they reached beyond.
- **Back Scratch Test (BST)**: The test assessed upper body flexibility. Participants stood and reached one hand over their same shoulder, palm facing down and fingers extended, reaching down the back as far as possible. They then tried to reach the other hand around their waist, palm facing up, to touch the first hand. The distance between the middle fingers of both hands was recorded: negative if they could not touch, zero if they touched, and positive if they overlapped.
- **Body Mass Index (BMI)**: This is a measure of weight relative to height, calculated by dividing weight in kilograms by height in meters squared.

#### Assessment of Sarcopenia Severity

**Fig. 1:**
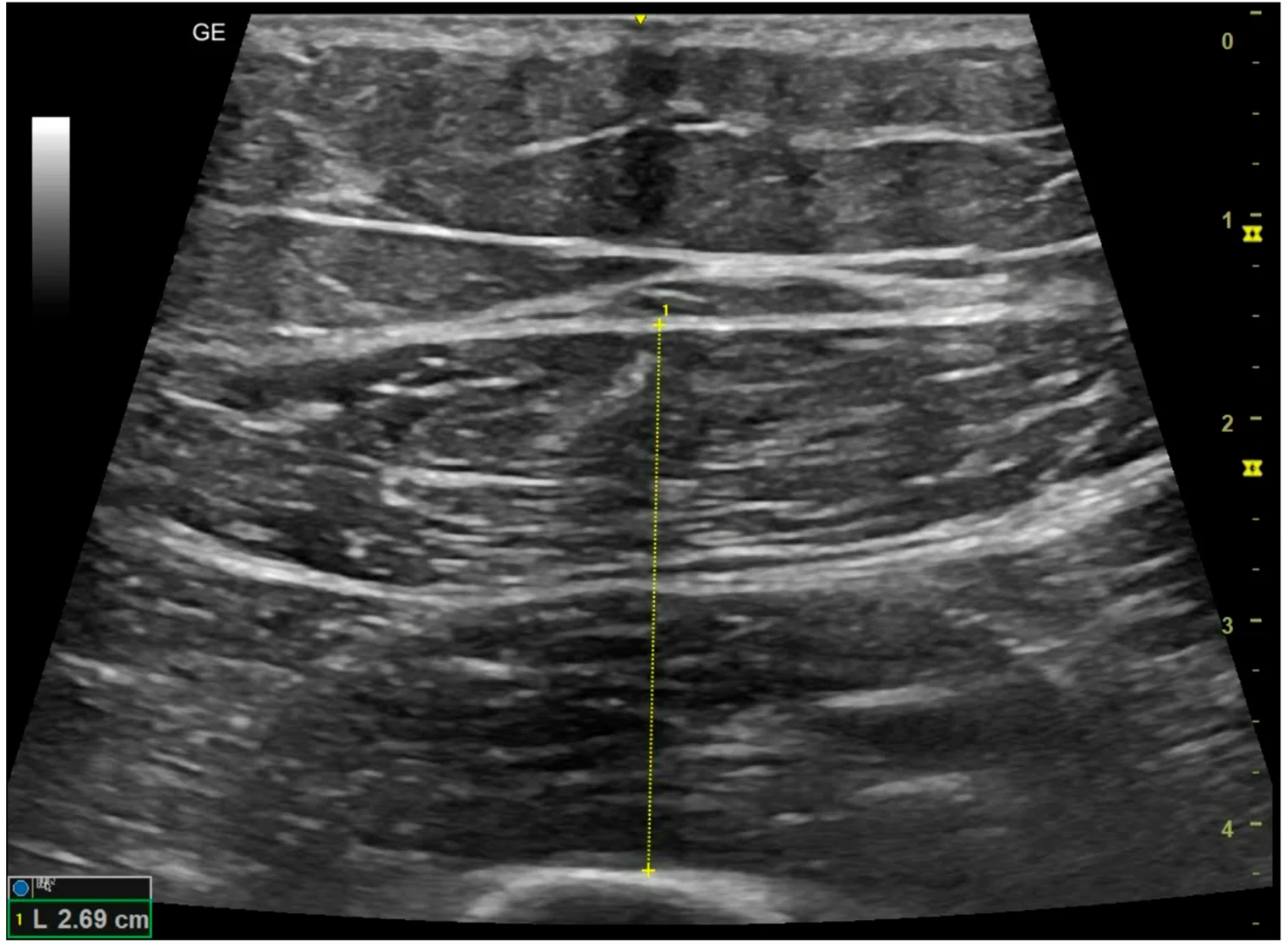
Axial Ultrasound Images (Anterior Thigh Thickness Measurement)

- **5-repetition Chair Stand Test (5r-CST)**: The 5r-CST measures the time it takes for an individual to stand up and sit down 5-repetitions as quickly as possible, starting from a sitting position without using their arms. A strength/power test such as the 5r-CST is important in the early diagnosis of sarcopenia [17].
- **Hand Grip Strength (HGS)**: Handgrip strength is a strong predictor of future morbidity and mortality in older adults. In this test, the individual sits on a chair with their feet flat on the floor. The Jamar Hand Dynamometer was used for measurement. They are asked to squeeze the dynamometer strongly with their shoulder adducted and in neutral rotation, elbow flexed at 90 degrees, forearm neutral and supported, and wrist neutral. 3-repetitions are performed, and the average is taken [18].
- **Ultrasonographic Measurements**: For sarcopenia evaluation, anterior thigh muscle thickness was measured by ultrasonography (7–12 MHz Logiq P5, GE Medical System). While the individuals were in the supine position, US images were obtained without any compression using plenty of gel. The right anterior thigh thickness (ATT) of all individuals was measured from the same point in the axial view (Fig. 1). Ultrasonographic measurements were made by a physician with at least 5 years of experience in this field, using a linear probe. According to the STAR method developed by Kara et al. [5], the STAR score was obtained by dividing the front thigh muscle thickness by the BMI for each individual.
  $$STAR\;=\frac{Anterior\;thigh\;thickness\;\left(mm\right)}{BMI\left(kg/m^2\right)}\;$$

Individuals were divided into dynapenic, sarcopenic, and non-sarcopenic according to the ISarcoPRM diagnostic algorithm [19]. According to this algorithm, the criteria were as follows:

- **Sarcopenia criteria:** (1) individuals with a STAR score below 1.0 in women and below 1.4 in men, (2) individuals with a 5r-CST score of more than 12 s, and (3) individuals with a hand grip strength (HGS) of less than 32 kg in men and less than 19 kg in women.
- **Dynapenia criteria:** (1) individuals with a STAR score above 1.0 in women and above 1.4 in men, (5) individuals with a 5r-CST of more than 12 s, and (6) individuals with an HGS of less than 32 kg in men and less than 19 kg in women.
- **Non-sarcopenia criteria:** (1) individuals with a STAR score above the cut-off values, (5) Individuals with a 5r-CST of less than 12 s, and (6) individuals with an HGS of more than 32 kg in men and more than 19 kg in women.

### Statistical analysis

Statistical analyses were conducted using IBM SPSS Statistics 26. It was examined whether the data were normally distributed or not with the Shapiro-Wilk test and it was determined that our data were not parametric. Therefore, descriptive statistics were given with median and minimum-maximum values for numerical data. In addition, mean and standard deviation values were presented in the tables so that the data could be understood more easily. Qualitative variables are displayed with frequency and percentage values. The Mann-Whitney U test was used to compare groups, and Fisher’s exact test was used for categorical variables.

## Results

The mean age, gender, mental status, and exercise habits were similar between the groups (*p* > 0.05). However, the drug use of individuals in nursing home residents was higher than community-dwelling older adults (*p* < 0.05) (Table 1).

**Table 1:** Demographic and clinical characteristics of the older adults

		Nursing Home Residents (*n* = 51)		Community-Dwelling Older Adults (*n* = 52)		*p* ^a^
		$\:\overline{\mathbf{X}}\:$± SD	*\widetilde X* (min–max)	$\:\overline{\mathbf{X}}\:$± SD	*\widetilde X* (min–max)	
Age (year)		73.13 ± 7.01	70 (65–85)	72.53 ± 6.08	72 (65–84)	0.706
BMI (kg/m ^2^)		25.8 ± 2.75	26.5(20.5–29.3)	26.65 ± 2.47	26.8(20.9–29.9)	0.127
MMSE		25.3 ± 0.97	25 (24–27)	25.21 ± 1.24	25 (24–28)	0.363
		n	%	n	%	p^b^
Gender	Female Male	33 18	64.7 35.3	26 26	50 50	0.164
Medication	≥Two One None	43 8 0	84.3 15.7 0	27 16 9	51.9 30.8 17.3	< 0.001*
Regular Exercise Habit	Exercising None	35 16	68.6 31.4	28 24	53.8 46.2	0.158

a: Mann-Whitney U Test, b: Fisher’s Exact Test,$\:\overline{\mathrm{X}}$± SD: Mean ± Standart Deviation, *\widetilde X*(min-max): Median (minimum-maximum), kg: kilogram, m: meter, n: Number of patients, BMI: Body Mass Index, MMSE: Mini-Mental State Examination

In our study, it was seen that individuals in nursing home residents and community-dwelling older adults showed a homogeneous distribution in terms of sarcopenia severity (*p* > 0.05). When the groups were compared, the ATT, STAR, HGS and 5r-CST values of nursing home residents were lower than community-dwelling older adults (*p* < 0.05) (Table 2).

**Table 2:** Comparison of individuals in terms of sarcopenia severity

	Nursing Home Residents (*n* = 51)		Community-Dwelling Older Adults (*n* = 52)		*p* ^b^
	*n*	%	*n*	%	
Non-Sarcopenic Dynapenic Sarcopenic	8 9 34	15.7 17.6 66.7	16 7 29	30.8 13.5 55.8	0.117
	$\:\overline{\mathbf{X}}\:$± SD	*\widetilde X* (min–max)	$\:\overline{\mathbf{X}}\:$± SD	*\widetilde X*(min–max)	p^a^
ATT (mm)	27.25 ± 5.51	25.78(19.83–42.62)	29.90 ± 5.80	28.95(20.07–39.67)	0.008*
STAR	1.05 ± 0.18	0.97 (0.84–1.54)	1.11 ± 0.18	1.10 (0.80–1.46)	0.027*
HGS	18.64 ± 8.05	17.13 (7.90–33.67)	22.24 ± 7.49	19.86(11.78–42.43)	0.017*
5r-CST	15.93 ± 3.36	14.71 (10.01–23.43)	12.97 ± 2.16	12.80 (9.60–17.42)	< 0.001*

a: Mann-Whitney U Test, b: Fisher’s Exact Test, *: *p* < 0.05,$\:\overline{\mathrm{X}}$± SD: Mean ± Standart Deviation, *\widetilde X*(min-max): Median (minimum-maximum), mm: milimeter, ATT: Anterior Thigh Thickness, STAR: Sonographic T high Adjustment Rate, HGS: Hand Grip Strength, 5r-CST: 5 repetition Chair Stand Test

When the groups were compared, the CST-30s, ACT, 2-MST, CSRT, and BST values of nursing home residents were lower than community-dwelling older adults (*p* < 0.05). The 8-FUGT duration was shorter in community-dwelling older adults than in nursing home residents (*p* < 0.05). The BMI was similar between the groups (*p* > 0.05) (Table 3).

**Table 3:** The senior fitness test of the older adults

	Nursing Home Residents (*n* = 51)		Community-Dwelling Older Adults (*n* = 52)		*p* ^a^
	$\:\overline{\mathbf{X}}\:$± SD	*\widetilde X* (min–max)	$\:\overline{\mathbf{X}}\:$± SD	*\widetilde X*(min–max)	
CST-30s	9.84 ± 2.02	11 (7–13)	10.96 ± 2.15	11 (7–15)	0.029*
ACT	13.31 ± 5.33	13 (5–26)	16.78 ± 4.13	17 (9–26)	< 0.001*
8-FUGT (sec)	14.46 ± 3.69	14.71(6.67–20.40)	10.31 ± 1.98	10.58 (7.02–14.20)	< 0.001*
2- MST	54.45 ± 13.35	52 (31–90)	66.53 ± 13.40	68 (42–98)	< 0.001*
CSRT (cm)	−1.80 ± 6.41	0 ((−18) – 6)	1.48 ± 6.65	2 ((−18) – 11)	0.031*
BST (cm)	−18.98 ± 8.37	−20 ((−42) – 4)	−14.88 ± 10.27	−18 ((−31) – 17)	0.032*

a: Mann-Whitney U Test, *: *p* < 0.05,$\:\overline{\mathrm{X}}$± SD: Mean ± Standart Deviation, *\widetilde X*(min-max): Median (minimum-maximum), mm: milimeter, cm: centimeter, sec: second, CST-30s: Chair Stand Test (repetition/30 sec), ACT: Arm Curl Test (rep/30 sec), 8-FUGT: 8-Foot Up and Go Test (sec), 2-MST: 2-Min Step Test (repetition/2 min), CSRT: Chair Sit and Reach Test (distance-cm), BST: Back Scratch Test (distance-cm)

## Discussion

In this study, a new algorithm developed by experts in the special interest group on sarcopenia (ISarcoPRM) was used to determine the severity of sarcopenia in older adults [5]. This is the first study to compare the sarcopenia severity of nursing home residents and community-dwelling older adults with a new and updated algorithm using ultrasonographic measurement. The study results showed that physical fitness levels were lower in nursing home residents than in community-dwelling older adults. Although the groups were homogeneous in terms of the number of sarcopenic, dynapenic and non-sarcopenic individuals, it was observed that the ATT, STAR, HGS, and 5r-CST scores of individuals living in nursing homes were lower, meaning that the severity of sarcopenia was higher.

The groups were homogeneous in terms of age, BMI, cognitive level, gender and regular exercise habits. We believe that the higher rate of drug use in individuals living in nursing homes is due to the fact that individuals stay in nursing homes due to chronic diseases.

There are various tests in the literature to evaluate physical fitness in older adults. In a review previously conducted by Galhardas et al., physical and motor fitness tests used for older adults showed heterogeneity [14]. Many tests used to evaluate the physical fitness of frail geriatric individuals are easy tests and cannot fully evaluate physical fitness [2, 14, 20, 21]. The Senior Fitness Test was preferred in our study because it is a comprehensive test that evaluates physical fitness in older adults [22].

Sarcopenia evaluation is still discussed today. In a study conducted by Kara et al., they defined the STAR score to determine sarcopenia. In addition, in this study, cut-off values are given for methods such as HGS and 5r-CST, which are frequently used in the literature to determine sarcopenia [5, 7, 23]. In order to determine dynapenic and sarcopenic older adults a new algorithm based on ICF (International Classification of Functioning) developed by experts in the special interest group on sarcopenia (ISarcoPRM) was demonstrated in the study by Kara et al. [5].

There are a limited number of studies in the literature examining sarcopenia and physical fitness levels in nursing home residents and community-dwelling older adults. One of these is a cross-sectional study by Kazoglu et al. [2]. They compared the physical fitness levels of individuals living in the community and nursing home with the Senior Fitness test. This study showed that nursing home residents had better scores on the 2-MST and 8-FUGT than community-dwelling older adults. However, in Kazoglu et al.‘s study, no difference was observed between the groups in other parameters of the Senior Fitness Test. In our study results, community dwelling older adults had better scores on all parameters in the Senior Fitness Test than nursing home residents. The reason for these results may be that nursing home residents are dependent on caregivers in the institution where they live for their care and activities of daily living. In Kazoğlu et al. (2)’s study, the sarcopenia level of the individuals was not evaluated. The reason why we obtained different results in the study of Kazoğlu et al. [2] may be that individuals living in the community and individuals living in nursing homes are not distributed homogeneously in terms of sarcopenia.

When the literature is examined, the results of a systematic review study by Liu et al., showed that the prevalence of sarcopenia in nursing home residents varies between 25% and 73.7% while this rate varies between 5.2% and 62.7% in community-dwelling older adults [24]. The results of the study conducted by Senior et al. [25], examining the prevalence of sarcopenia in nursing home residents, support the results of Liu et al. [24].

In a review by Papadopoulou et al., they investigated the prevalence of sarcopenia in hospitalized geriatric individuals, nursing home residents, and community-dwelling older adults. It was shown that the prevalence of sarcopenia was higher in hospitalized geriatric individuals and nursing home residents [26]. When the literature was examined, it was seen that bioelectrical impedance analysis was generally used in the methodology in studies comparing the sarcopenia prevalence of older adults. However, the fact that we used the ISarcoPRM diagnostic algorithm [5] developed for the diagnosis of sarcopenia in our study and that it also diagnosed dynapenic older adults while comparing sarcopenia severity is one of the strengths of our study.

The inclusion of geriatric individuals in a single city in our study, the use of a 2-minute step test due to the environment in which we worked, and the fact that geriatric individuals are not suitable for the 6-minute walk test may be limitations of our study.

In conclusion, our study results showed that geriatric individuals living in nursing homes had higher sarcopenia levels and lower physical fitness levels than community-dwelling older adults. The physical fitness levels should be maintained in nursing home residents. Regular physical activity programs can be recommended for older adults. We achieved these results because they are dependent on the caregivers in the institution they live in for their self-care and daily life activities.

## Data Availability

The data of this study are available.
